# Complication de prothèse intermédiaire

**DOI:** 10.11604/pamj.2015.21.295.7137

**Published:** 2015-08-24

**Authors:** Redouane Ouakrim, Mohamed Saleh Berrada

**Affiliations:** 1 Service de Chirurgie Orthopédique, CHU de Rabat, Rabat, Maroc

**Keywords:** Prothèse intermédiaire, arthrose, hanche, Interim prosthesis, arthrosis, hip

## Image en medicine

La prothèse intermédiaire de hanche est la technique la plus fréquemment utilisée dans le cadre de fracture du col très déplacée. L'articulation de la hanche n’étant pas arthrosique, seule la partie fémorale est remplacée par la prothèse. En cas d'arthrose ancienne lors de la fracture du col du fémur, il va falloir opter pour une prothèse totale de hanche. La technique d'intervention chirurgicale est la même que celle d'une prothèse totales de hanches. Le type d'incision et la voie d'abord varient en fonction de la technique utilisée. Nous rapportons le cas d'une patiente de 82 ans qui a présente une complication mécanique 5 ans après la pose de sa PIH. Cette complication peut se voir chez des malade ostéoporotique, d'où l'intérêt de surveiller de prêt cette population de patient au moins une fois par an.

**Figure 1:**
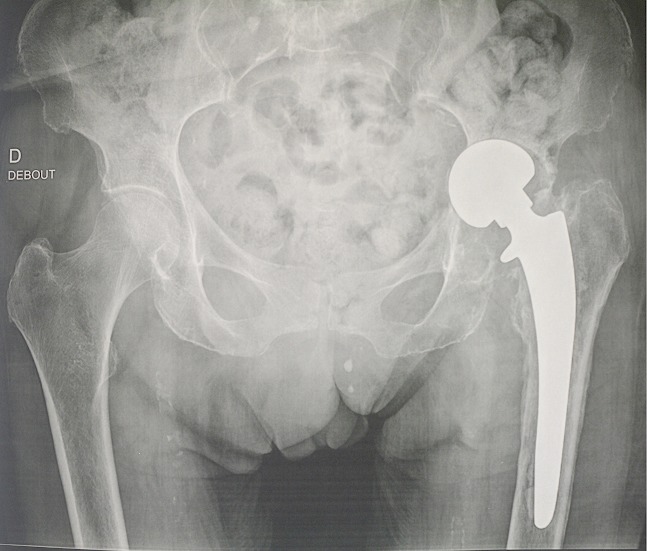
cotyloïdite suite à une PIH

